# Nutrition for Infant Feeding

**DOI:** 10.3390/nu14091823

**Published:** 2022-04-27

**Authors:** Alessandra Consales, Daniela Morniroli, Giulia Vizzari, Fabio Mosca, Maria Lorella Giannì

**Affiliations:** 1 Department of Clinical Sciences and Community Health, University of Milan, 20122 Milan, Italy; alessandra.consales@unimi.it (A.C.); daniela.morniroli@unimi.it (D.M.); fabio.mosca@unimi.it (F.M.); 2 Fondazione IRCCS Ca’ Granda Ospedale Maggiore Policlinico, NICU, 20122 Milan, Italy; giulia.vizzari@unimi.it; 3 Società Italiana di Nutrizione Pediatrica, 20126 Milan, Italy

It has long been demonstrated that nutrition in the first 1000 days of life can affect health outcomes later in life. Indeed, according to the Developmental Origins of Health and Disease (DOHaD) hypothesis, the roots of several disorders manifesting in childhood and adult life can be traced back to the prenatal and early infant phases. In particular, an ever-growing body of literature agrees on identifying the early post-natal period as an extremely sensitive time-window for nutritional programming. Consequently, infant feeding is now recognized as one of the main modifiable risk factors for long-term health outcomes.

Unsurprisingly, research on the long-term effects of infant diet is rapidly expanding. The eight papers (five original research articles, two reviews and one study protocol) contained in this Special Issue focus on the influence of nutrition, from lactation to complementary feeding, on infants’ health, with the intention of updating knowledge on the impact of specific feeding strategies on anthropometric growth, body composition, neurofunctional development, metabolic processes and protection against infections through the first years of life.

The prospective study conducted in Amsterdam by Sirkka et al. [1] examined the impact of various infant feeding factors on the BMI of children of Dutch and Turkish descent at 2, 3 and 5 years of age. Through their notable work, the Authors found some interesting and not easily explained age-dependent ethnic inequalities in childhood BMI.

The study by Guzzardi et al. [2] investigated the role of early feeding practices in cognitive development in preschool children and found a strong positive association between exclusive breastfeeding in the first 6 months of life and hearing and language scores in 5-year-old girls. The Authors attentively reviewed the current literature and carefully avoided potential biases that flawed previous studies conducted on the subject. Preschool children were chosen as the age group of interest to avoid the potential influence of school education and social stimuli on cognitive development. Possible confounders were rigorously considered, such as objectively measured parental IQ and maternal and offspring’s BMI. Furthermore, children who had been exclusively breastfed were considered separately from all the others, so as to guarantee a more homogeneous study sample.

Kouwenhoven et al. [3] conducted an interesting multi-centre, double-blinded RCT investigating if and how a 20% reduction in protein intake could alter the metabolic and hormonal profile at the age of 4 months in term infants fed a low-protein formula with a unique customized blend of essential amino acids compared to infants fed a standard formula and breastfed infants. The Authors also assessed the long-lasting association between specific blood markers and body composition and anthropometric parameters until 2 years of age. To avoid potential biases deriving from the introduction of solid foods, parents were requested to delay weaning until after 17 weeks of age.

In their experimental cohort study, nested within a multi-centre factorial RCT, Muelbert et al. [4] used NIRS to assess changes in oxygenated hemoglobin (O2Hb) concentrations in the orbitofrontal cortex of moderate-to-late-preterm newborns before and during gastric tube feeds in a clinical setting. Muelbert’s original study paves the way for future research investigating the effect of sensory stimulation preceding tube feeds on clinical outcomes, as well as the impact on cortical activation of feedings in a NICU setting.

Analyzing data from the National Health and Nutrition Examination Survey (NHANES), Young et al. [5] sought an answer to whether and how variation in macronutrient ingredients within the standard formula category may affect infant growth. Through their results, the Authors remind us that not all infant formulas are the same!

The review by Cerasani et al. [6] explores recent advances in the knowledge of the effects of breast milk on growth and body composition in premature infants, highlighting the importance of the promotion and support of human milk feeding also in this population. Indeed, despite a slower weight gain compared to formula feeding, human milk promotes fat-free mass deposition, thus favoring a better recovery of body composition and ultimately leading to improved metabolic and neurodevelopmental outcomes.

The review by Morniroli et al. [7] focuses on a topic that has become increasingly more appealing, especially during these latest pandemic times: the antiviral properties of breast milk. Morniroli effectively underlines the polyvalent nature of breast milk, a unique functional food for the infant, and prompts fellow researchers to keep exploring all its apparently endless possibilities.

Finally, the paper by Shek et al. [8] elegantly describes an RCT study protocol evaluating the impact on infant growth and body composition of a concept infant formula containing lipid droplets closely mimicking those in human milk. Importantly, the study will apply an innovative breastfeeding-friendly cohort-like recruitment approach, randomizing study participants based on the parents’ autonomous decision to start (or not) formula feeding during the study period.

In conclusion, the original articles and reviews included in this Special Issue are likely to substantially aid our understanding of the influence of early nutritional exposure on infants’ health and promote the identification of effective feeding strategies to improve growth and developmental outcomes. The advances presented in this Special Issue are of great interest from a clinical perspective and may hopefully act as the basis for future research on this fascinating topic.

## Abbreviations

BMI: body mass index; DOHaD: developmental origins of health and disease; IQ: intelligence quotient; NIRS: near-infrared spectroscopy; RCT: randomized controlled trial.
